# Pregnancy planning health information and service needs of women with chronic non-communicable conditions: a systematic review and narrative synthesis

**DOI:** 10.1186/s12884-022-04498-1

**Published:** 2022-03-22

**Authors:** Karin Hammarberg, Ruby Stocker, Lorena Romero, Jane Fisher

**Affiliations:** 1 Global and Women’s Health, School of Public Health and Preventive Medicine, Monash University, Melbourne, Australia; 2 The Ian Potter Library, The Alfred, Melbourne, Australia

## Abstract

**Background:**

Preparing for pregnancy and being in the best possible health before conception improves reproductive outcomes. For women living with a chronic non-communicable disease (NCD), pregnancy planning is essential to allow optimal disease control in preparation for pregnancy.

**Aim:**

The aim was to review the literature relating to the pregnancy planning health information and service needs of women with NCDs.

**Method:**

The MEDLINE (Ovid), Embase (Ovid), Emcare (Ovid), PsycINFO (Ovid), CINAHL and Scopus databases were searched. Studies were included if they were published in peer-reviewed English language journals between January 2010 and June 2020 and reported on the pregnancy planning health information and service needs of women with rheumatic diseases, asthma, cystic fibrosis, depression and/or anxiety, type 1 diabetes mellitus, epilepsy, or multiple sclerosis. Risk of bias was assessed using QualSyst. The characteristics of the studies were tabulated and summarised. Key findings of the included studies were analysed thematically using an inductive approach, where the study findings determined the themes. Findings are reported in a narrative synthesis.

**Results:**

The database searches yielded 8291 results, of which 4304 remained after duplicates were removed. After abstract screening 104 full-text papers were reviewed. Of these 15 met inclusion criteria and were included in analysis. The narrative synthesis of the included studies revealed six themes: ‘Women with chronic conditions have unmet preconception health information needs’, ‘Women with chronic conditions want personalised preconception health information’, ‘Preferred sources of preconception health information’, ‘Learning from the experiences of other women’, ‘Improving preconception health discussions with health care professionals’, and ‘Women want holistic care’. These themes were consistent across all studies, highlighting the similarity of experiences and needs of women with different chronic conditions.

**Conclusion:**

To improve pregnancy outcomes for women living with NCDs, health care providers need to ask women of reproductive age proactively and routinely about their pregnancy intentions and provide them with personalised advice on how to avoid unplanned pregnancy and be in optimal health when they wish to conceive.

PROSPERO registration number CRD42020176308.

**Supplementary Information:**

The online version contains supplementary material available at 10.1186/s12884-022-04498-1.

## Background

Parental preconception health optimisation is emerging as an important population health and disease prevention strategy. Maternal and paternal obesity, poor nutrition, smoking, excessive alcohol consumption, poor mental health, and recreational drug use are all potentially modifiable factors that are associated with poorer pregnancy outcomes [1, 2]. It is known that during the periconception period, the time from maturation of gametes through to early embryonic development, parental health and health behaviours influence offspring health at birth and their long-term risks of cardiovascular, metabolic, immune, and neurological morbidities [3]. Experts argue that preconception care should be offered to women and their partners planning pregnancy and that system-wide public health interventions are needed to optimise the health of all women and men of reproductive age [2]. They suggest that preconception health promotion should be offered opportunistically in all clinical encounters with women of reproductive age and that women planning pregnancy should receive individualised preconception care based on their health, health behaviours, and unique needs [4, 5]. Preconception care involves a range of strategies including counselling regarding substance use in pregnancy, advice about diet and folic acid and iodine supplementation, supporting weight reduction in those who are overweight or obese, adjusting medication if required, ensuring immunisations are up-to date, and screening for sexually transmitted infections and other infectious diseases [6].

Pregnancy planning and avoiding unintended pregnancy are key to allowing people to take steps to be as healthy as possible before they try to conceive. The One Key Question® (OKQ) concept developed in the US proposes that women of reproductive age should be asked routinely “Would you like to become pregnant in the next year?” in primary healthcare encounters [7]. Depending on their answer, they should then be provided with patient-centred advice, which is tailored to their desire for, wish to avoid, or ambivalence about pregnancy. This would entail ensuring that women who want to avoid pregnancy have reliable contraception and informing those who desire or are ambivalent about pregnancy of the benefits of optimal preconception health and encouraging them to seek preconception care before trying to conceive [8]. Similarly, the Reproductive Life Planning (RLP) concept recommends that discussions about women’s reproductive intentions and contraceptive practices and needs are integrated into women’s routine healthcare to reduce the risk of unintended pregnancies and help women achieve planned and well-timed pregnancies [5]. Public health experts and health professional organisations also promote opportunistically including questions about pregnancy intention in primary care settings to improve awareness about the importance of preconception health [6, 9–12].

There are common barriers to asking about pregnancy intention and promoting preconception health in primary care [13–17]. These include lack of knowledge, skills, and resources to initiate conversations about optimising health before conception; lack of time; and that the sensitive nature of the topic prevents health professionals from having preconception health conversations with their patients. Findings from other studies suggest that there are opportunities to improve preconception health awareness through education, social media campaigns, and within healthcare systems [18] and that most people would not mind being asked about their pregnancy plans by their healthcare provider [19].

Non-communicable diseases (NCDs) are long lasting conditions with persistent effects on health. To manage their condition, people living with NCDs often require medication and regular contact with medical specialists and allied health professionals. Maternal NCDs are known to adversely affect reproductive outcomes and the proportion of women with chronic conditions giving birth is increasing [20]. For women living with NCDs, pregnancy decision making is more complex than for other women as they need to consider the additional potential risks of pregnancy on their own health and the heath of the fetus [21].

For women living with NCDs, pregnancy planning is essential to allow optimal disease control in preparation for pregnancy, changing a potentially teratogenic treatment regimen to one that is safer for the fetus, and contraceptive advice to delay or avoid pregnancy until it is desired, and the woman is in the best possible health [4].

To inform clinical care, the aim was to review the literature relating to the pregnancy planning health information and service needs of women with chronic non-communicable health conditions.

## Methods

### Data sources and searches

The systematic review was conducted according to the PRISMA guidelines [22]. The search strategy was designed by a specialist information analyst to find published studies in 6 databases MEDLINE (Ovid), Embase (Ovid), Emcare (Ovid), PsycINFO (Ovid), CINAHL and Scopus. After an initial search for articles in Medline and Embase, an analysis of the text words contained in the title and abstract, and of the index terms used to describe these articles was conducted. A second search using identified key words and index terms was then undertaken from January 2010 to July 2020 across all six databases. The search strategies used a combination of Subject Headings and free text terms that aimed to cover the topic areas of [1] Pre-pregnancy, preconception, peri-conception planning AND [2] Information and knowledge needs or preferences, AND [3] Chronic or non-communicable or non-infectious diseases or conditions. Searches were adapted as appropriate to the specifications of each of the 6 databases. The final search is presented in the supplementary file.

The search strategy, inclusion criteria and analysis method were specified in advance and documented in a protocol registered with PROSPERO [23].

### Study inclusion

Qualitative and quantitative primary research studies published in peer-reviewed English language journals between January 2010 and June 2020 which reported on the pregnancy planning health information and service needs of women with rheumatic diseases (RD), asthma, cystic fibrosis (CF), depression and/or anxiety, type 1 diabetes mellitus (T1D), epilepsy, or multiple sclerosis (MS) were included. The included conditions were chosen after comparing various population reports of NCDs affecting women of reproductive age and a systematic review conducted a decade ago [24]. Editorials, reviews, and studies investigating menopause, sexually transmitted infections, pregnancy care, or abortion care were excluded.

### Data extraction and quality assessment

The search results were exported into Covidence. Two authors (RS and KH) independently completed title and abstract screening of all search results. Discrepancies were discussed and resolved. The same two authors screened the full-text articles against the inclusion and exclusion criteria.

Data from the included studies were extracted and compiled in a table, using Excel. The data extracted were: study design, study methodology, study setting, number of participants, participant characteristics (e.g. age), chronic health condition, outcome and outcome measure (if applicable), and main findings.

Risk of bias was assessed using QualSyst developed by Kmet et al. [25]. The QualSyst assessment tool provides a systematic reproducible and quantitative means of assessing the quality of both quantitative and qualitative studies. Possible QualSyst scores range from 0.0 to 1.0.

### Data analysis and synthesis

The characteristics of the studies were tabulated and summarised. Key findings of the included studies were analysed thematically using an inductive approach, where the study findings determined the themes. After repeated reading of the papers, study findings were coded, and themes identified. The thematic structure was finalised, and its meaning interpreted through discussion among researchers. Findings are reported in a narrative synthesis. Narrative synthesis is more than a summary of findings as it allows exploration of similarities and differences between studies. It ‘ … *refers to an approach to the systematic review and synthesis of findings from multiple studies that relies primarily on the use of words and text to summarise and explain the findings of the synthesis’ (p 5)* [26].

## Results

### Search results

**Fig. 1:**
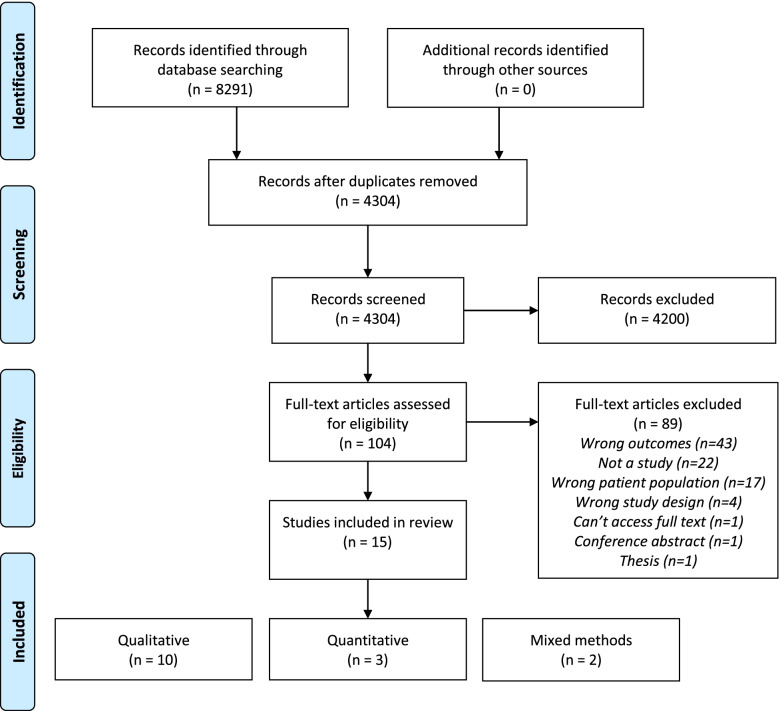
PRISMA flowchart

The database searches yielded 8291 results, of which 4304 remained after duplicates were removed (Fig. 1). At the title and abstract screening stage, 4200 studies were excluded, leaving 104 full-text papers which were assessed for eligibility. Eighty-nine studies did not meet the inclusion criteria and were excluded, mainly because the research question or outcomes did not relate to preconception health information (*n* = 89), they were not an empirical study (*n* = 22) or had the wrong patient population (*n* = 17). Ultimately, 15 studies met the eligibility criteria and were included in this review.

### Study characteristics

The study characteristics and main findings are described in Table 1.

**Table 1:** Study characteristics and main findings

Author (year); country	Aim	Study design	Inclusion criteria; Recruitment	Data collection tools	Data analysis	Participant characteristics	Main findings relevant to pregnancy planning and preconception health
**Rheumatic disease**							
Ackerman et al. (2015); Australia	Determine the information needs of women with RD concerning pregnancy, post-natal care and early parenting	Mixed methods; *Quant:* questionnaire; *Qual:* telephone interviews and focus groups, sampled for maximum heterogeneity	Women with RD, aged 18–45, taking disease-modifying antirheumatic drugs and who have been pregnant in the last five years, currently pregnant or planning to become pregnant in the next five years; Advertised through arthritis consumer organisations, peer support groups and via health professionals	*Quant:* questionnaire; Educational Needs Assessment Tool (ENAT)^a^ Autonomy Preference Index (API) ^b^ *Qual:* interview guide	*Quant:* descriptive *Qual:* thematic, inductive and deductive	*n* = 27 (*n* = 15 telephone interviews, *n =* 12 focus groups); median age 32 yrs.; median 5 yrs. since diagnosis; 13/27 resided in a major city; *n* = 18 pregnant in the last 5 yrs., *n* = 5 currently pregnant, *n =* 18 considering pregnancy in the next 5 yrs.	• *Quant:* o High educational needs (mean (SD) ENAT score 97.2 (30.8)) o API showed need for information exceeds what is required for decision making • *Qual themes*: o Lack of specific pregnancy information o Information needs dependent on individual circumstances including educational level and occupation o Rheumatologist primary information source but online forums and social media also used o Information gaps identified; women want decision-making support, to learn from other women o Want accessible online information o Suggest arthritis consumer organisations can be resource hubs.
Briggs et al. (2016); Australia	Establish cross-discipline consensus on the educational messages and practice behaviours in the management of women with RD relating to contraception, pregnancy, breast feeding and early parenting	Qualitative; eDelphi study	Rheumatologists, obstetricians, and pharmacists with ≥4 yrs. experience, currently practicing in Australia for at least 8 h/week; Self-nomination, personal invitation from researchers, snowball sampling, advertising at meetings and conferences	Three rounds R1: 10 free-text questions about the information needs of women with RD R2 and 3: synthesis of themes and level of agreement with information	Thematic, inductive	*n* = 36 (*n =* 22 rheumatologists, *n =* 9 obstetricians, *n* = 5 pharmacists)	• Final set = 18 themes, supported by 5 meta-themes • Support/strong support for key themes ranging from 88.2–100% • Meta-themes: o Coordination in information delivery o Mode and timing of information delivery o Evidence-based information o Engagement of the right HCP o Non-judgemental approaches • For conception and pregnancy: Important to achieve optimal disease control prior to considering pregnancy - encourage early discussions.
Chew et al. (2019); Global (online)	A qualitative study of threads on Reddit, to understand the pregnancy related information needs and concerns of women with RD	Qualitative; analysis of publicly available Reddit threads	Threads from 2008 to 2018, relating to arthritis, pregnancy, and parenting, from the subreddits ‘r/Thritis’, ‘r/Rheumatoid’, and ‘r/BabyBumps’	Reddit threads	Thematic	59 threads (*n* = 16 r/Thritis, *n* = 34 r/Rheumatoid, *n* = 9 r/BabyBumps)	• Themes: o Finding a community o Making decisions about pregnancy o Worrying about the impacts of arthritis on pregnancy and parenting o Information needs for managing arthritis throughout the perinatal period o Seeking pregnancy information and resources for women with arthritis.
Phillips et al. (2018); UK	Identify the information and support needs of women with autoimmune rheumatic diseases (ARD) during pregnancy planning, pregnancy and early parenting	Mixed methods; *Quant:* online survey *Qual:* individual interviews and free text responses from survey	*Quant:* women with ARD, 18–49 yrs., planning pregnancy, pregnant now or in the last 5 yrs., and/or has child < 5 yrs. *Qual:* women, as above and HCPs caring for women with ARDs; Study website, social media, patient organisations, peer support groups, professional networks	*Quant:* online survey; ENAT^a^, Arthritis Impact Measurement Scale V 2 Short Form^c^ *Qual:* Women: flexible narrative approach, assisted by a visual timeline and free-text responses from survey; HCPs: interview guide on role of caring for women with RD	*Quant:* descriptive *Qual:* thematic, deductive and inductive	*Quant: n* = 128 *Qual: n =* 22 women interviewed, *n* = 118 provided free-text comments in survey; *n* = 7 HCPs interviewed	• *Quant:* o High educational needs (ENAT mean score 104.9 ± 30.18), greatest about preparing for and increasing chance of pregnancy o Want peer support (63%), alternative/complementary therapies (56%), care coordination (52%) • *Qual themes*: o Information needs o Multi-disciplinary management o Accessing support - especially peer support • Want timely, high quality, accessible information about pregnancy planning, disease activity and management • HCPs recognise unmet information need and importance of pregnancy planning.
Wolgemuth et al. (2020); USA	Explore the sexual and reproductive health care and counselling needs of young women with RD in the context of their rheumatology care	Qualitative; semi-structured individual interviews	Women aged 18–45 yrs. with RD; Potentially eligible patients at outpatient rheumatology clinics in Pennsylvania, invited to participate	Interview guide	Grounded theory, inductive	*n* = 30; mean age 35.1 yrs.; *n* = 14 had no children, *n =* 1 pregnant	• Want rheumatologists to initiate conversations about SRH at first visit and to revisit at subsequent visits • Want clear and complete information from rheumatologists about risks of and to pregnancy, particularly risks of medication • Want SRH considered in the context of their life, personal values and rheumatic disease • Feel like intermediaries between rheumatologists and gynaecologists • Want HCPs to recognise that pregnancy plans may change over time.
**Cystic fibrosis**							
Holton et al. (2019); Australia	To identify the childbearing concerns and related information needs and preferences of women with cystic fibrosis (CF)	Qualitative; online discussion in a private Facebook group	Women with CF aged ≥18 yrs. who speak English and live in Australia; Recruited through Facebook ads, and engagement with Australian CF organisations’ Facebook pages	Discussion guide to initiate and prompt discussion, moderator added a new question every few days; Anonymous online survey to collect demographic characteristics and fertility history	Thematic	*n =* 11 (only 9 posted comments); mean age 32.2 yrs.; all had post-secondary qualifications; most (9/11) in a relationship; mean number of children 0.8 (range 0–3)	• Use CF HCPs and other women with CF as sources of information • Hard to find and access relevant, accurate and up-to-date information about CF and childbearing • Want personalised information based on medical evidence and personal experiences, particularly about medication during pregnancy, and physical risks of pregnancy on CF • Want information from puberty, or prior to needing it (pre-pregnancy), and regular pregnancy and fertility discussion.
Kazmerski et al. (2017a); USA	To explore attitudes and decision-making regarding pregnancy among young women with CF	Qualitative; semi-structured individual interviews	Women with CF aged 18–30 yrs., attending an inpatient or outpatient visit at an adult CF care centre	Interview guide, assessing attitudes toward fertility/pregnancy and experiences with preconception counselling and reproductive care	Thematic, iterative	*n =* 22; mean age 25.1 yrs.; CF severity *n =* 9 mild, *n =* 10 moderate, *n =* 3 severe; *n =* 5 pregnant once before, *n =* 2 pregnant twice or more	• CF a major factor in reproductive decision-making • Confusion about how CF can affect fertility/pregnancy. • Perceived disapproval from CF care providers regarding pregnancy • Dissatisfaction with reproductive care in the CF setting • Want early and improved SRH education, repeated and open discussions initiated by CF providers.
Kazmerski et al. (2017b); USA	To investigate the attitudes and practices of CF providers toward SRH care in young women with CF	Quantitative; anonymous online survey	CF providers (physicians, nurse practitioners (NPs) and physician assistants (PAs)) in the US; Advertised through US CF program directors and centre officials, networks of the Cystic Fibrosis Foundation, and informal CF provider social media sites	Online survey, with multiple choice questions about attitudes towards SRH care, current SRH care practices, preferences for SRH care in CF, barriers and facilitators to SRH in the CF care model	Descriptive, logistic regression	*n* = 196; 24% physicians caring for adults, 57% physicians caring for children, 19% NPs/PAs; mean age 47.8 yrs.; 48% male	• 94% agreed SRH is important/very important for young women with CF • 75% thought SRH care should be standardised in the CF care model • 40% believed CF team have the primary role in SRH discussions • 90% rated planning for pregnancy as important/very important but only 59% discussed it at least yearly • > 90% thought the CF team should initiate SRH discussions • Barriers for discussing SRH: lack of time, presence of others, lack of knowledge, patient discomfort, lack of rapport, provider discomfort • Enablers for discussing SRH: provider training, partnership with SRH specialists, guidelines, sessions about SRH at conferences, access to educational resources for patients and families.
**Diabetes (type 1)**							
Edwards et al. (2016); Australia	To describe and develop a model of the pregnancy journey for women with type 1 diabetes (T1D)	Qualitative; analysis of conversations between women with diabetes and Diabetes Counselling Online (DCO)	Conversations between women with T1D and DCO between 2002 and 2012	Electronic conversations between women with T1D and DCO	Thematic	*n* = 93 individual conversations, 200 interactions (1–44 interactions per woman)	• 7 phases of the pregnancy journey identified: contemplation, pre-pregnancy planning, conception, pregnancy, loss, delivery and birth, motherhood • 3/7 themes relevant to contemplation and pre-pregnancy planning o Balance and juggling (what to eat, how much to exercise etc) o Impact of diabetes on pregnancy (baby’s and own health) o Knowledge seeking and application (wanting to know what to do to be in best possible health)
Grady & Geller (2016); USA	To examine the relationship between locus of control, self-efficacy, and outcome expectations of preconception counselling in women with T1D	Quantitative; online survey	Women with T1D, aged 18–44 yrs., with no previous pregnancy; Recruited through advertising on websites relevant to women with T1D, support forums, social media	Reproductive Health Attitudes and Behaviour instrument, Diabetes-Specific Locus of Control measure and study-specific socio- demographic and diabetes/pregnancy questions	Descriptive, multiple regression	*n* = 147; mean age 25.88 yrs.; 76.9% desired future pregnancy; mean duration of T1D 14.13 ± 8.68 yrs	• Only 44.9% correctly listed at least three health risks for pregnant women or fetus of poorly controlled blood glucose • 76.2% had never received formal preconception counselling from a HCP • 34% had asked for pregnancy information • Self-efficacy for planning a healthy pregnancy was positively associated, and self-blame about disease management negatively associated, with perceived usefulness of preconception counselling.
McCorry et al. (2012); UK	To explore attitudes toward pregnancy planning and preconception care seeking in women with diabetes	Qualitative; semi-structured individual interviews	Women with T1D, aged 18–40 yrs., in the South Eastern Health and Social Care Trust area in Northern Ireland; Eligible women attending a participating hospital invited in writing	Interview guide developed from a literature review and research team discussion	Thematic, iterative	*n =* 14; median age 28 yrs.; median 11 yrs. since diagnosis; *n* = 4 had children	• Themes: o Emotional complexity of childbearing decisions o Preferences for information relating to pregnancy o Being known by your health professional o Frustration with medical model of care • Anxiety about pregnancy commonly because of limited knowledge • Many did not understand why pregnancy planning is important • Want detailed information to help with pregnancy decision-making rather than just being told to have ‘good control’.
Paiva et al. (2016); Portugal	To explore the views of women with T1D in relation to preconception care	Qualitative; semi-structured individual interviews	Women with T1D attending the APDP - Diabetes Portugal clinic in Lisbon; Recruited through the APDP clinic’s database	Interview guide; demographic data also collected	Phenomeno-logical	*n* = 6; age range 24–45 yrs.; *n =* 2 had a previous pregnancy (1 live birth)	• Themes: o Fear of complications o Information o Communication approach o External support o Autonomy • Women were unsure about what to do when planning a pregnancy • Information from HCPs or self-sourced often perceived as conflicting or ad hoc • Want information on preconception diabetes management • Want more supportive communication from HCPs, and personalised information.
Spence et al. (2010); UK	To determine knowledge and attitudes of women with T1 and T2 diabetes of childbearing age towards pre-pregnancy care	Qualitative; focus group discussions	Women with T1D or T2D, aged 16–40 yrs., English speaking; Recruited through outpatient records at two National Health Service hospitals	Short demographic questionnaire; interview guide with open-ended questions	Content analysis	Four focus groups: (A) young nulliparous women with T1D, (B) older nulliparous women with T1D, (C) parous women with T1D, (D) women with T2D of mixed parity; *n* = 24 (*n =* 18 with T1D)**;** age range 17–40 yrs.; diabetes duration 2–33 yrs.; *n =* 5 ever received pre-pregnancy advice	• Themes: o Knowledge o Quality of relationships with HCPs - positive and negative, often conflicting, discouraging o Organisation of care o HCPs attitudes affect their advice o Women’s attitudes to pre-pregnancy care advice • Generally aware of importance of pregnancy planning, but not sure why or what issues to focus on • GP, nurse and consultant, internet sources of information. Want to learn from other women with diabetes • Lack of continuity of care • HCPs stereotyping of pregnancy, parenting, marriage, etc. a perceived barrier to seeking PC care.
**Epilepsy**							
Friedrich, Sruk & Bielen (2018); Croatia	To explore the knowledge, sources and needs for information regarding pregnancy-related issues in epilepsy (PRIE)	Quantitative; anonymous online survey	Women treated for epilepsy, aged 15–45 yrs.; Advertised to all visitors to the Croatian Association for Epilepsy website and Facebook page	Questionnaire: demographics, pregnancy and epilepsy questions, knowledge about PRIE, prior information about pregnancy and epilepsy	Descriptive, multiple regression analysis	*n* = 200; mean age 29 yrs.; 35% had previous pregnancy, 43% desired children in the future	• Knowledge test (range 0–5) mean score 3.50 ± 1.27 • Having been counselled by a neurologist, and higher use of books/brochures predicted better knowledge • Less than half had been counselled on pregnancy by their neurologist • Need for PRIE information greater than the information provided • Of women who had discussed pregnancy with neurologist, 68% had themselves initiated the conversation • 61% preferred verbal information from neurologist and 22% preferred written information.
**Multiple sclerosis**							
Kosmala-Anderson & Wallace (2013); UK	To explore the experiences, and assess the expectations and needs of women with MS, in relation to childbearing	Qualitative; semi-structured individual interviews	Women with MS who attended neurology clinic in the previous three months for planned, current or recent pregnancy; Recruited from the neurology clinic, given invitation and study information by an MS nurse; opportunistic sampling	Interview guide; women asked about their experience of discussing pregnancy with HCPs, pregnancy information and support needs	Thematic, inductive	*n =* 9; mean age 30.6 yrs.; *n =* 3 considering pregnancy, *n* = 3 currently pregnant, *n* = 3 recently given birth; 8/9 had relapsing-remitting MS	• Themes: o Concerns about MS and pregnancy o Lack of information about MS and pregnancy o Others’ opinions about childbearing • All reported difficulty finding information about MS and childbearing • Wanted to know about impact of medication on fertility, miscarriage, how long before conception to stop medication • Wanted to know about MS symptoms during pregnancy and risk of relapse after • All had discussed pregnancy with a HCP, all discussions initiated by the women.

CF cystic fibrosis, HCP health care professional, MS multiple sclerosis, PRIE pregnancy-related issues in epilepsy, RD rheumatic disease, SRH sexual and reproductive health, T1D type 1 diabetes, T2D type 2 diabetes

^a^Hardware B, Anne Lacey E, Shewan J. Towards the development of a tool to assess educational needs in patients with arthritis. Clin Eff Nurs. 2004;8 [2]:111–7

^Ende J, Kazis L, Ash A, Moskowitz MA. Measuring patients’ desire for autonomy: decision making and information-seeking preferences among medical patients. J Gen Intern Med. 1989;4 [1]:23–30

^c^Guillemin F, Coste J, Pouchot J, Ghézail M, Bregeon C, Sany J. The AIMS2-SF: a short form of the Arthritis Impact Measurement Scales 2. French Quality of Life in Rheumatology Group. Arthritis Rheum. 1997;40 [7]:1267–74

#### Chronic conditions

The included studies reported on women with five chronic conditions: RD (*n* = 5) [27–31], cystic fibrosis (*n* = 3) [32–34], T1D (*n =* 5) [35–39], and one each on epilepsy [40] and MS [41]. There were no studies on the needs for preconception health information of women with asthma, depression, or anxiety.

#### Participants

In 11 of the 15 studies, women with a chronic condition were the participants [27, 30–32, 34, 36–41]. Most of these studies included a mix of women who had never been pregnant and women who were currently or had previously been pregnant. There were some exceptions: Grady and Geller [36] only included women who had never been pregnant and McCorry et al. [37] excluded women if they were currently pregnant.

One study [29] analysed threads from the social media platform Reddit, where users participate in online discussions. As Reddit is an anonymous platform, individual demographic and disease information could not be obtained. Thus, although this study specifically searched rheumatoid arthritis and pregnancy-related threads, we cannot be sure that all participants were women with rheumatoid arthritis. Two studies [28, 34] solely involved health care providers (HCPs) as the participants, and one mixed-methods study [30] involved HCPs as well as women with chronic conditions.

Spence et al. [39] included women with both type 1 and type 2 diabetes. However, data were disaggregated which allowed us to extract the data that pertained to the women with T1D.

#### Study setting

The included studies were conducted in six different countries: three in Australia [27, 28, 32], one in Croatia [40], one in Portugal [38], four in the United Kingdom [30, 37, 39, 41], four in the United States [31, 33, 34, 36], and two were global (online-based) [29, 35].

#### Study design and data sources

Ten studies used qualitative methods, three were quantitative, and two used mixed methods. Of the qualitative studies, five involved semi-structured individual interviews, and one, focus group discussions; all these studies used an interview guide to aid the discussion. One study was a 3-round eDelphi study to establish consensus recommendations. The three other qualitative studies analysed data from online fora and social media groups.

The three quantitative studies were all online cross-sectional surveys. The two studies using mixed methods employed similar methods; the quantitative component was a survey, and the qualitative component involved individual interviews or focus group discussions. Both mixed-method studies’ surveys included the Educational Needs Assessment Tool (ENAT) [42], one included the Autonomy Preference Index [43], and the other the Arthritis Impact Measurement Scale Version 2 Short Form [44]. In both mixed methods studies, a study-specific interview guide was used in the qualitative component.

### Quality assessment

Overall, the included studies were of a high quality as measured by QualSyst. For the qualitative studies and qualitative components of the mixed methods studies, the mean score was 0.81 (range 0.7–0.95). The criterion that was most infrequently addressed was reflexivity; seven of the 12 studies made no comment on reflexivity, four made a partial attempt, and only one study had a full reflexivity statement. The mean score for the quantitative studies was 0.93 (range 0.86–1.0) (Supplementary material).

### Main findings

The narrative synthesis of the included studies revealed six themes. These themes were consistent across all studies, highlighting the similarity of experiences and needs of women with different chronic conditions.

#### Women with chronic conditions have unmet preconception health information needs

Overall, this body of literature shows that women with a chronic condition have unmet needs relating to preconception health information. Several studies showed that although women are aware that preconception health and pregnancy planning are important, they are not sure why [37, 39].

*The thing they most wanted to tell me about at this age (20 years) was to use some form of contraception, because you don’t want to get pregnant without it being planned … but they don’t say why it needs to be planned.* (Woman with T1D) [39].

Women’s information needs appear to be bi-directional; they want to know about the impact of their chronic condition on preconception health, pregnancy and motherhood, and also about the impact of pregnancy on their chronic condition. In terms of the first direction, women want to know how they can best prepare for pregnancy in the context of their chronic condition. In particular, they want to know about the risks associated with medications they are taking, and how to prepare for changes in medication that might be needed before they conceive. In Ackerman et al.’s mixed methods study [27], women with RD reported that the area in which they needed most information was regarding the toxicity of medication and its impact on the fetus and breastfed baby. Women also want more information about how pregnancy might affect their chronic condition and the symptoms associated with it [29], and have practical concerns about being pregnant [28].

The consequences of having unmet information needs were discussed in some papers [27, 34, 37]. In McCorry et al.’s qualitative study [37], women with T1D described that their anxiety surrounding pregnancy was largely due to a lack of knowledge about the impact of pregnancy on their condition, and how this influenced their pregnancy-related decision making.

*It’s just like the unknown ... you need information, so that you can make the decision in your own way, ‘cause everybody’s different.* (Woman with T1D) [37].

Evidence also suggests that some HCPs may not be well-equipped to provide preconception health advice. In a survey of HCPs caring for women with CF, they acknowledged the importance of discussing sexual and reproductive health (SRH) with women with CF. However, 44% of participants stated that their lack of knowledge was a barrier to having such discussions. Most agreed that training modules, conferences, and partnerships with SRH specialists would improve their knowledge and capacity to discuss pregnancy intention and provide preconception health advice to their patients [33].

#### Women with chronic conditions want personalised preconception health information

Studies showed that women want individualised preconception health information tailored to their condition and circumstances [27, 31, 32, 38]. In the qualitative component of Ackerman et al.’s [27] mixed-method study, a theme emerged about the lack of condition-specific information on pregnancy planning, pregnancy and early parenting for women with RD. Women described how arthritis is often perceived as a condition that only affects older people, and that information materials frequently only include images of elderly people. Women with CF identified the need for personalised information, as the severity of CF can vary considerably between women [32]. In Paiva et al.’s study [38], women with T1D also described their desire for information to be more personalised and adapted to their current pregnancy intentions.

#### Preferred sources of preconception health information

Actual and preferred sources of PCH information was investigated in some studies. Findings indicate that women’s main source of information about preconception health and pregnancy planning is their chronic condition specialist; for example, their rheumatologist [27], neurologist [40], or endocrinologist [39]. In Friedrich et al.’s study [40], nearly two thirds of women with epilepsy reported that they prefer to receive information personally from their neurologist.

The lack of PCH information appears to drive women to seek information from informal sources. In online discussions between women with CF, participants described how it can be difficult to find and access relevant, accurate and updated information about CF and childbearing [32].

*I feel there is very limited information and you really have to work hard to find it! I follow blogs on Facebook (of mothers with* CF *who have had child/ren), [and] have consulted with my* CF *team.* (Woman with CF) [32].

Women’s preference for more coordinated delivery of PCH information was evident. In Wolgemuth et al.’s study [31], women with rheumatic diseases reported that they often feel like intermediaries between their rheumatologists and obstetrician-gynaecologist. In other studies, the value of multidisciplinary care was emphasised both by women with chronic conditions and HCPs [30, 41].

#### Learning from the experiences of other women

Women with chronic conditions were also found to have a strong wish to connect with other women in similar situations. A recurring theme throughout the studies was that, in addition to wanting information and medical advice about PCH from HCPs, women want to learn from the experiences of women with similar chronic conditions who have had children. In Chew et al.’s [29] analysis of Reddit threads relating to pregnancy and parenting, the importance to women with RD of finding a community was evident. Chew et al. [29] describe how knowing other women with the same chronic condition enables opportunities for sharing concerns, asking about experiences, giving and receiving social support, and gaining self-motivation. In the quantitative component of one mixed methods study, 62.5% of women with RD agreed that they would appreciate peer support [30]. In their analysis of written interactions with an online counselling support service Edwards et al. found that peer support offers the most reassurance for women with T1D contemplating pregnancy [35]. Based on this, they recommend that pregnancy-related resources for women with T1D should include accounts of the lived experiences of pregnancy planning, pregnancy, and motherhood of women with T1D. Interestingly, when HCPs who care for women with RD participated in an eDelphi study to establish consensus on pregnancy related educational messages and best practice behaviours in the management of women with RD, peer support was only advised in the early parenting period [28].

#### Improving PCH discussions with HCPs

Some studies explored the characteristics of PCH discussions that are important to women. They revealed that women want discussions about PCH to start early (e.g. in puberty) and be repeated [31, 32, 34]. It was also evident across many studies that women want pregnancy-related conversations to be initiated by HCPs, to encourage more open and trusting discussions [31, 34]. Additionally, in one study more than 90% of CF physicians, nurse practitioners and physician assistants agreed that the CF team should initiate sexual and reproductive health conversations with women of reproductive age [33]. This contrasts with the experiences of those who had previously had a PCH discussion. In Friedrich et al.’s [40] survey of women with epilepsy, 68% of those who had discussed pregnancy or breastfeeding with their neurologist had raised the topic themselves. Similarly, in Kosmala-Anderson & Wallace’s [41] qualitative study, all women with MS who were interviewed had initiated discussions about pregnancy intentions with the HCP.

Women in some studies emphasised that their pregnancy plans are likely to change over time, and that they therefore want HCPs to ask them about their pregnancy intention more than once [31, 32]. In other studies women remarked that HCPs’ personal opinions might explain why they do not ask about pregnancy intention or discuss PCH; women with CF reported that many perceived disapproval from CF providers regarding pregnancy [34] and women with T1D felt that the timing of advice from HCPs indicated stereotypical views about marriage or childbearing.

*The second they spotted an engagement ring, they said “Are you planning a pregnancy?”! Well, maybe I would have had one before I was engaged. Like I’m 27, so … just because there’s an engagement ring on my finger doesn’t mean all of a sudden I’m going to have a baby. I might have had one before that!* (Woman with T1D) [39].

In response to this, Spence et al. recommend that HCPs ask all women of reproductive age about their pregnancy intentions, irrespective of their relationship or marital status [39].

#### Women want holistic care

The final theme that emerged is the strong desire for women with chronic conditions to receive more holistic care. Women with RD reported wanting their sexual and reproductive health and their chronic condition to be considered in the broader context of their life circumstances and personal values [31].

*I think people when they get so focused on their specialty, sometimes they’re not thinking about how that can affect other aspects of someone’s life … Thinking holistically about their approach to an individual’s care, they need to consider other parts of their life, mental, physical, and what your plans are, what your life is about.* (Woman with RD) [31].

Women with T1D in McCorry et al.’s [37] study shared their desire for HCPs to recognise the complexity of managing diabetes within the context of their life.

*I want them to recognize that there’s a lot of life that gets in the way of things and complex areas—and they don’t realize those complexities, so that’s why I feel a little bit frustrated. I also think that sometimes doctors need to be aware that pregnancy for some women is a roller coaster.* (Woman with T1D) [37].

## Discussion

This review identified that women living with chronic conditions have unmet preconception health information and service needs and that there are ways in which this can be improved. Considering the significant effect of preconception health on the health of women and their offspring, health care professionals in all settings, including general practitioners and medical specialists, have a shared responsibility to help women to be as healthy as possible before they conceive.

Limitations of the review include that only a limited number of NCDs and outcomes were included and that there was no process for external validation of the themes we identified.

Overall, the methodological quality of the included studies was high. The small number of studies identified in the search and the lack of studies of the preconception health information needs of women diagnosed with asthma, anxiety, and depression indicate this is an under-researched area and that more research is needed. While the themes identified in the review were consistent across all included studies, it cannot be presumed that they apply to women with chronic conditions not covered in this review. To gauge what is known about the preconception health information needs of women with chronic conditions not included in this review, a future more comprehensive review of the literature is warranted.

The women who participated in the reviewed studies were self-selected and inclusion criteria for most were that women were considering pregnancy, were pregnant, or had children. Findings should therefore be interpreted with caution as they do not represent women with chronic conditions who are unable to or choose not to have children. They may resent being asked repeatedly about pregnancy intention during routine healthcare consultations. To avoid causing distress, clinicians need systems that identify the women for whom questions about pregnancy intention are inappropriate.

The review revealed that women with chronic conditions want to know how their condition might affect a pregnancy and how a pregnancy might affect their condition; want health care providers to initiate discussions about pregnancy intention from adolescence and for this to recur throughout their reproductive years as pregnancy plans may change over time; and that they rely on their specialist as their main source of information about preconception health and pregnancy planning. These findings indicate that asking women of reproductive age whether they wish to have children should be an integral part of a consultation, irrespective of their age or relationship status. The One Key Question and Reproductive Life Planning concepts may help health professionals ask about reproductive intention and provide the advice women need either to avoid unintended pregnancy or be informed about the benefits of planning pregnancy to ensure that their medical condition is well managed, and they are as healthy as possible when they conceive. Health professionals who interact with women of reproductive age with chronic conditions in other settings, for example general practitioners, obstetricians, nurses, and midwives should also promote pre- and inter-conception health messages when the opportunity arises.

While pregnancy may be risky for women with some chronic conditions, this does not prevent desire for pregnancy and parenthood. However, evidence indicates that women sometimes encounter disapproval from health professionals when they voice a wish to have a child. Rather than presuming that women with complex conditions do not want children, women want their health care providers to offer holistic, non-judgemental, well-coordinated, multidisciplinary care to reduce pregnancy-related risks and improve the health outcomes for them and their babies. In addition, there is some evidence that condition-specific decision aids can improve knowledge about a chronic condition in the context of pregnancy planning and reduce decisional conflict for women contemplating pregnancy [45, 46].

As has been found in studies of primary health care providers [15, 16], this review found that specialists are aware of the importance of discussing sexual and reproductive health with women of reproductive age but report that their lack of knowledge and skills are a barrier to doing this. This is supported by the finding that almost all the women who had discussed pregnancy intention and PCH with their specialist, had initiated this discussion. Training opportunities, including online learning programs and collaboration with sexual and reproductive health experts or obstetricians might improve specialists’ knowledge and capacity to discuss pregnancy intention and provide condition-specific preconception health advice to their patients.

Across the studies, it was evident that women with chronic conditions, who contemplate pregnancy, value peer support and want to hear the stories of women with the same condition who have been pregnant and given birth. Narrative health messages where people with lived experience share their stories in video-based education are the most effective in terms of modifying complex behaviour [47, 48]. This suggests that condition-specific videos with women sharing their experiences of pregnancy planning, pregnancy and motherhood would be powerful educational tools for women with chronic non-communicable diseases who consider pregnancy.

In conclusion, to improve pregnancy outcomes for women living with NCDs, the health care professionals who care for them need to ask women of reproductive age proactively and routinely about their pregnancy intentions and provide them with personalised advice on how to avoid unplanned pregnancy and be in optimal health when they wish to conceive. Based on the findings of this review, the preconception health information and service needs of women with chronic conditions are not met, in part because of the lack of knowledge about this aspect of health promotion among health care providers. The review identified potential strategies to rectify this including training and educational resources to improve health care providers’ capacity to discuss pregnancy plans with women in a non-judgemental way; a holistic approach to discussing the potential risks of pregnancy considering the woman’s unique circumstances; multidisciplinary care; and women having access to peer support and the narratives of peers who have lived experience of pregnancy planning.

## Supplementary Information

**Additional file 1.**

**Additional file 2.**

## Data Availability

All data generated or analysed during this study are included in this published article and its supplementary information files.
