# Supplementary material for: Pregnancy planning health information and service needs of women with chronic non-communicable conditions: a systematic review and narrative synthesis
Source: BMC Pregnancy Childbirth. 2022 Mar 22;22:236. doi: 10.1186/s12884-022-04498-1 (PMC8941766; doi:10.1186/s12884-022-04498-1)
Supplement: Supplementary file 1 — Additional file 1. [file 12884_2022_4498_MOESM1_ESM.docx]

Database(s): Ovid MEDLINE(R) and Epub Ahead of Print, In-Process & Other Non-Indexed Citations, Daily and Versions(R) 1946 to July 06, 2020 Search Strategy:

| **#** | **Searches** |
| --- | --- |
| 1 | Preconception Care/ or reproductive health services/ or family planning services/ or Reproduction/ or Reproductive Behavior/ or Reproductive Medicine/ or Reproductive Health/ or reproductive techniques/ or reproductive techniques, assisted/ or fertility preservation/ or Fertility Clinics/ or fertility/ or ovarian reserve/ or infertility/ or infertility, female/ or fertility agents/ or exp fertility agents, female/ |
| 2 | exp Contraception/ or Contraception Behavior/ or reproductive control agents/ or contraceptive agents/ or exp contraceptive agents, female/ or exp contraceptive agents, hormonal/ or contraceptive devices/ or exp contraceptive devices, female/ |
| 3 | (pre-pregnancy or prepregnancy or preconception* or pre-conception* or periconception* or peri-conception* or (family planning or reproductive health*) or ("try* to conceive" or "plan* to conceive" or "capacity to conceive" or "ability to conceive" or before conception or "prior to conception" or becoming pregnant or (trying to become pregnant or wanting to get pregnant or wanting to conceive))).mp. |
| 4 | (((pregnan* or conception) adj3 (plan* or intention* or intend* or prepar* or decision*)) or (reproducti* adj3 (concern* or decision* or intention* or plan or plans or planning))).mp. |
| 5 | (birth control or fertility control or antifertility or anticonceptive* or Infecundity or subfertility or hypofertility or "interval to conception" or "interval to pregnancy" or "time to conception" or "time to pregnancy" or "struggl* to conceive" or female steril* or reproductive* steril* or ((utili#e* or utili#ation* or utili#ing or "use" or "us?ing") adj1 contracepti)).mp. |
| 6 | ((future adj (family or pregnanc* or children or conception or reproducti*)) or (children adj3 (plan* or intention* or intended or desir*) adj3 (have or having))).mp. |
| 7 | (((childbearing or child-bearing or reproductive) adj (age or years)) or (reproductive adj (issue* or desire*))).mp. |
| 8 | ((fertility or infertility or infertile or fecundity) adj3 (concern* or decision* or intention* or plan or plans or planning or issue* or desire* or support*)).mp. |
| 9 | or/1-8 |
| 10 | communication/ or access to information/ or health communication/ or information dissemination/ or information literacy/ or information seeking behavior/ or health education/ or consumer health information/ or health literacy/ or patient education as topic/ or communication barriers/ or Counseling/ or Health Knowledge, Attitudes, Practice/ |
| 11 | decision making/ or choice behavior/ or uncertainty/ or problem solving/ or decision making, shared/ or decision support techniques/ or Health Behavior/ or cooperative behavior/ or empowerment/ or help-seeking behavior/ or Self Care/ |
| 12 | "health services needs and demand"/ or needs assessment/ or Health Services Accessibility/ or attitude to health/ or "patient acceptance of health care"/ or patient participation/ or patient satisfaction/ or patient preference/ or professional-patient relations/ or nurse-patient relations/ or physician-patient relations/ |
| 13 | (((unmet or met or meet*) adj4 (need* or want*)) or ((assess* or satisf* or identif*) adj3 (need* or demand*))).mp. |
| 14 | ((information* or advice or instruct* or knowledge or counsel* or opinion* or wisdom or guidance or recommendations) adj3 (need* or seek* or sought or requir* or search* or given or receiv* or availab* or perception* or perceive* or desir* or want* or asking or demand* or satisf* or dissatisf*)).mp. |
| 15 | (((awareness or enlightenment or comprehension or direction or discussion or answers) adj1 (need* or want* or require* or seek* or sought)) or ((learning or education* or health literacy) adj need*)).mp. |
| 16 | (((know or answer* or learn) adj2 (need* or want* or demand*)) or ((understanding or communication) adj2 (need* or want* or require* or seek* or sought or access*))).mp. |
| 17 | (((locat* or obtain* or access* or lack* or gather* or sourc*) adj3 (information or knowledge)) or ((utili#e* or utili#ation* or utili#ing or "use" or "us?ing") adj3 information)).mp. |
| 18 | ((information* adj (priorit* or behavio?r* or interest? or resource* or booklet* or leaflet* or pamphlet* or brochure*)) or ((information or communication or knowledge) adj2 (type or types or mode or modes or receipt or barrier* or challeng* or appropriat* or retain* or written or verbal*)) or (misinform* or disinform*)).mp. |
| 19 | ((knowledge* or information*) adj2 (updating or up-to-date or timely or develop* or gain* or limited or prefer* or provide* or provision or distribution or delivery or lack* or gap or gaps or level? or understand* or question* or help* or share* or sharing or attitude* or interpret* or perceiv* or perception*)).mp. |
| 20 | ((psycholog* or psychosocial* or emotional or health* or medical or help or care or support or consultation* or service* or specialist*) adj2 (need* or want* or seek* or sought or prefer* or desiring or access* or require* or demand* or access*)).mp. |
| 21 | (((utili#e* or utili#ation* or utili#ing or "use" or "us?ing") adj1 (health* or care or service* or support)) or ((choice* or choose or choosing or decision* or attitude*) adj2 (health* or care or service* or support))).mp. |
| 22 | (patient* adj1 (educat* or knowledge or inform* or teach* or advice or advising or choice* or decision* or empower* or view* or experience* or support*)).mp. |
| 23 | (wom#n* adj (need* or want* or desire* or prefer*)).mp. |
| 24 | (((consumer* or patient* or client* or customer*) adj4 (need* or want* or desire*)) or ((consumer* or patient* or client* or customer*) adj3 (interest* or prefer* or satisf* or perspective* or experience* or attitude* or belief* or behavio?r* or practice* or concern? or support* or participat* or advoca* or focus* or empower* or expectation* or opinion* or view* or perceiv* or perception* or involv* or priorit*))).mp. |
| 25 | (self efficacy or self management or self care or self help or ((information or education or knowledge) adj intervention*)).mp. |
| 26 | or/10-25 |
| 27 | chronic disease/ or multiple chronic conditions/ or (noncommunicable diseases/ and chronic*.mp.) |
| 28 | (chronic health condition* or chronic* ill* or chronic medical condition* or chronic disease* or chronic* sick*).mp. |
| 29 | ((chronic and (non-communicable or non-infectious or noncommunicable or noninfectious)) adj (disease* or condition* or illness*)).mp. |
| 30 | exp Multiple Sclerosis/ or exp Arthritis/ or exp Epilepsy/ or exp Diabetes Mellitus, Type 1/ or Cystic Fibrosis/ or exp Asthma/ |
| 31 | mood disorders/ or depressive disorder/ or depressive disorder, major/ or depressive disorder, treatment-resistant/ or dysthymic disorder/ or seasonal affective disorder/ or cyclothymic disorder/ or anxiety disorders/ or agoraphobia/ or neurotic disorders/ or obsessive-compulsive disorder/ or panic disorder/ or phobic disorders/ |
| 32 | (depressive neuros#s or depressive syndrome or endogenous depression or melancholia or neurotic depression or unipolar depression or anxiety disorder* or depressive disorder* or mood disorder* or affective disorder* or dysthymic disorder* or neurotic disorder* or obsessive-compulsive disorder* or panic disorder* or phobic disorder* or cyclothymic disorder* or neurotic anxiety or phobic anxiety or phobic neuros#s).mp. |
| 33 | (Multiple Scleros#s or Arthrit* or Polyarthrit* or Periarthrit* or Spondylarthrit* or Osteoarthrit* or Epilep* or seizure disorder* or Cystic Fibros#s or Mucovisc?idosis or fibrocystic disease* or Asthma* or Type 1 Diabetes or autoimmune diabetes or insulin-dependent diabetes or juvenile-onset diabetes or ketosis-prone diabetes or sudden-onset diabetes or diabetes type 1 or T1DM or IDDM or ketoacidotic diabetes or juvenile diabetes).mp. |
| 34 | Depression/dt or Anxiety/dt |
| 35 | exp antirheumatic agents/ or exp anticonvulsants/ or exp antidepressive agents/ or exp anti-anxiety agents/ or exp anti-asthmatic agents/ |
| 36 | or/27-35 |
| 37 | 9 and 26 and 36 |
| 38 | (female* or wom#n* or girl*).mp. |
| 39 | 37 and 38 |
| 40 | ((pre-pregnancy or prepregnancy or preconception* or pre-conception* or periconception* or peri-conception* or pregnancy planning or family planning or fertility or infertil* or contraception or contraceptive* or reproductive health) adj3 (knowledge or information or communication or education or discussion* or informed choice* or counsel* or advice or decision* or choice* or option*)).mp. |
| 41 | ((pre-pregnancy or prepregnancy or preconception* or pre-conception* or periconception* or peri-conception* or pregnancy planning or family planning or fertility or infertil* or contraception or contraceptive* or reproductive health) adj3 (need* or seek* or support* or access* or service* or care or resource* or practice* or experience* or aware* or perception* or understanding or views)).mp. |
| 42 | 40 or 41 |
| 43 | 36 and 38 and 42 |
| 44 | 39 or 43 |
| 45 | limit 44 to english language |
| 46 | 45 not (hiv or human immunodeficienc* or human immun* deficienc*).mp. |
| 47 | limit 46 to (case reports or comment or editorial or letter or news or newspaper article) |
| 48 | 46 not 47 |
| 49 | limit 48 to yr="2010 -Current" |
