# Supplementary material for: Pregnancy planning health information and service needs of women with chronic non-communicable conditions: a systematic review and narrative synthesis
Source: BMC Pregnancy Childbirth. 2022 Mar 22;22:236. doi: 10.1186/s12884-022-04498-1 (PMC8941766; doi:10.1186/s12884-022-04498-1)
Supplement: Supplementary file 2 — Additional file 2. [file 12884_2022_4498_MOESM2_ESM.docx]

Kmet scoring for Qualitative studies

| **Author (year)** | **1. Question/objective sufficiently described?** | **2. Study design evident and appropriate?** | **3. Context for the study clear?** | **4. Connection to a theoretical framework/wider body of knowledge?** | **5. Sampling strategy described, relevant and justified?** | **6. Data collection methods clearly described and systematic?** | **7. Data analysis clearly described and systematic?** | **8. Use of verification procedure(s) to establish credibility? *NO PARTIAL** | **9. Conclusions supported by the results?** | **10. Reflexivity of the account?** | **Final score** |
| --- | --- | --- | --- | --- | --- | --- | --- | --- | --- | --- | --- |
| Chew et al. (2019) | Yes | Yes | Yes | Yes | Yes (threads searched, not all were relevant) | Yes | Yes | No | Yes | No | 0.8 |
| Ackerman et al. (2016); qual component | Yes | Yes | Partial | Yes | Yes | Yes | Yes | Yes | Yes | No | 0.85 |
| Briggs et al. (2016) | Yes | Yes | Yes | Yes | Yes | Partial | Partial | Yes | Yes | No | 0.8 |
| Phillips et al. (2018); qual component | Yes | Yes | Yes | Yes | Partial | Yes | Yes | Yes | Yes | Yes | 0.95 |
| Wolgemuth et al. (2020) | Yes | Yes | Partial | Yes | Yes | Partial | Yes | Yes | Yes | No | 0.8 |
| Holton et al. (2019) | Yes | Yes | Yes | Yes | Yes | Partial | Partial | No | Yes | No | 0.7 |
| Kazmerski et al. (2017a) | Yes | Yes | Yes | Yes | Partial | Yes | Yes | No | Yes | No | 0.75 |
| McCorry et al. (2012) | Yes | Yes | Yes | Yes | Yes | Yes | Yes | Yes | Yes | Partial | 0.95 |
| Edwards et al. (2016) | Yes | Partial | Partial | Yes | Partial | Yes | Yes | Yes | Partial | Partial | 0.75 |
| Paiva et al. (2016) | Yes | Yes | Partial | Yes | No | Partial | Yes | Yes | Yes | No | 0.7 |
| Spence et al. (2010) | Yes | Yes | Yes | Yes | Yes | Yes | Yes | No | Yes | Partial | 0.85 |
| Kosmala-Anderson & Wallace (2013) | Yes | Yes | Yes | Yes | Partial | Yes | Yes | No | Yes | Partial | 0.8 |
|  |  |  |  |  |  |  |  |  |  |  |  |
|  |  |  |  |  |  |  |  |  |  | Mean | 0.81 |

Kmet scoring for quantitative studies

| **Author (year)** | **1. Question/objective sufficiently described?** | **2. Study design evident and appropriate?** | **3. Method of subject/comparison group selection OR source of information/input variables described and appropriate?** | **4. Subject (and comparison group, if applicable) characteristics sufficiently described?** | **5. If interventional and random allocation was possible, was it described?** | **6. If interventional and blinding of investigators was possible, was it reported?** | **7. If interventional and blinding of subjects was possible, was it reported?** | **8. Outcome and (if applicable) exposure measure(s) well defined and robust to measurement/misclassification bias? Means of assessment reported?** | **9. Sample size appropriate?** | **10. Analytic methods described/justified and appropriate?** | **11. Some estimate of variance is reported for the main results?** | **12. Controlled for confounding?** | **13. Results reported in sufficient detail?** | **14. Conclusions supported by the results?** | **Final score** |
| --- | --- | --- | --- | --- | --- | --- | --- | --- | --- | --- | --- | --- | --- | --- | --- |
| Ackerman et al. (2016); quant component | Yes | Yes | Yes | Yes | N/A | N/A | N/A | Yes | Partial | Yes | Yes | N/A | Yes | Yes | 0.95 |
| Kazmerski et al. (2017b) | Yes | Yes | Yes | Yes | N/A | N/A | N/A | Yes | Yes | Yes | N/A | Yes | Partial | Yes | 0.95 |
| Friedrich, Sruk & Bielen (2018) | Yes | Partial | Yes | Yes | N/A | N/A | N/A | Yes | Yes | Partial | Yes | Partial | Yes | Yes | 0.86 |
| Phillips et al. (2018); quant component | Yes | Yes | Yes | Yes | N/A | N/A | N/A | Yes | Yes | Yes | Yes | N/A | Yes | Yes | 1 |
| Grady & Geller (2016) | Yes | Yes | Yes | Yes | N/A | N/A | N/A | Yes | Yes | Partial | Yes | No | Yes | Yes | 0.86 |
|  |  |  |  |  |  |  |  |  |  |  |  |  |  | Mean | 0.93 |
